# The Corporation of London

**Published:** 1907-01-26

**Authors:** 


### THE CORPORATION OF LONDON.

### UTILISING THE BY-PRODUCTS IN ISLINGTON NEW ABATTOIR.

THE Corporation of the City of London have decided to equip the new abattoirs in course of construction at Islington with the best appliances, so that the by-products may be converted into a marketable commodity. They have placed with Messrs. William Douglas and Sons, Limited, of Putney, orders for two No. III. Podewils apparatuses complete, and these, it is estimated, will be equal to dealing with all the condemned carcasses and cognate by-products which may be produced. This equipment, which is common all over the Continent, is only beginning to be known in the United Kingdom. It converts every portion of a carcass into an impalpable powder, which can be used as a fertiliser or for cattle and fat.

